# NaviDiv: a web app for monitoring chemical diversity in generative molecular design

**DOI:** 10.1039/d5dd00487j

**Published:** 2026-03-30

**Authors:** Mohammed Azzouzi, Thanapat Worakul, Clémence Corminboeuf

**Affiliations:** a Laboratory for Computational Molecular Design, Institute of Chemical Sciences and Engineering, École Polytechnique Fédérale de Lausanne (EPFL) 1015 Lausanne Switzerland mohammed.azzouzi@epfl.ch; b National Center for Competence in Research-Catalysis (NCCR-Catalysis), École Polytechnique Fédérale de Lausanne (EPFL) 1015 Lausanne Switzerland

## Abstract

The rapid progress in generative models for molecular design has led to extensive libraries of candidate molecules for biological and chemical applications. However, ensuring these molecules are diverse and representative of broader chemical space remains challenging, with researchers often over-exploring limited regions or missing promising candidates due to inadequate monitoring tools. This work presents NaviDiv (Navigating Diversity in Chemical Space), a comprehensive web-based framework for managing chemical diversity in the string-based generative molecular design through three integrated capabilities: multi-metric diversity analysis capturing structural, syntactic, and molecular framework variations; interactive real-time visualization enabling immediate detection of model collapse; and adaptive constraint generation that dynamically guides optimization while preserving diversity. Through a singlet fission material discovery case study using REINVENT4, we demonstrate that different diversity metrics (*i.e.* structural similarity, fragment composition, and sequence patterns) respond differently during optimization, with constraint effectiveness depending critically on representational alignment with the generative model. *n*-Gram-based constraints outperform fingerprint-based approaches due to direct correspondence with SMILES generation, while combined constraints maintain diversity across all metrics while achieving optimization performance within 15% of unconstrained baselines. The framework is freely available at https://github.com/LCMD-epfl/NaviDiv, providing accessible tools for data-driven decisions about diversity–property trade-offs in automated molecular discovery.

## Abstract 2

The rapid progress in generative models for molecular design has led to extensive libraries of candidate molecules for biological and chemical applications.

## Graphical abstract

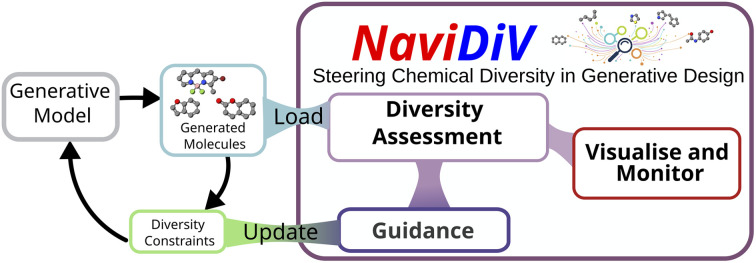

## 1 Introduction

Generative models represent a promising approach to molecular design, moving beyond the constraints of predefined molecular libraries^1–11^ or hand-crafted assembly rules.^12–14^ These models learn to represent molecules in high-dimensional latent spaces and are trained to reproduce the statistical distribution of chemical structures in the training data.^5,15^ By leveraging large and continuously expanding molecular databases, generative models can be trained to capture extensive chemical diversity and develop a data-driven understanding of molecular composition, thereby enabling the generation of novel compounds for specific applications.^16–18^

Generative molecular models are often employed for proposing molecules with targeted properties. To achieve this, a guidance strategy must be implemented to steer the generation, effectively shifting the distribution of generated molecules toward higher-performing candidates. The guidance approach differs from one model architecture to another; for example, gradient-based methods have been used on the latent space to optimise the property of interest in variational autoencoders (VAEs) and diffusion models.^19–21^ In the case of autoregressive models^22–25^ such as REINVENT,^26,27^ this is accomplished by updating model weights and biases through a policy-driven approach^28,29^ that optimizes the generation of molecules with properties aligned with specific criteria. This optimization is carried out *via* reinforcement learning, where actions involve adjusting model parameters, and the objective typically focuses on increasing the mean performance of the generated molecules. However, as the model is optimized, it inevitably focuses the generation on specific types of molecules in the confined region of the chemical space, leading to a reduction in molecular diversity. To mitigate excessive deviation from the initially trained model, a regularization term can be introduced within the reinforcement learning framework.^29^ Nevertheless, this regularization alone is often insufficient to prevent model collapse (loss of chemical diversity over successive training generations).^30^

Existing approaches to diversity preservation operate at different levels of the generative pipeline. Policy-based methods modify reinforcement learning strategies or employ multi-agent frameworks to enhance exploration.^26,27,31^ Others introduce diversity penalties at the evaluation function level, discouraging structurally similar molecules or recurring fragments.^32,33^ However, these approaches typically employ fixed parameters and single diversity metrics, lacking systematic monitoring of how constraints impact chemical space exploration during optimization. *Post hoc* diversity assessment methods^31,34–36^ enable comparison across generative models but cannot guide real-time intervention. This creates a critical gap: researchers can measure final diversity outcomes but cannot observe or respond to diversity loss as it occurs.

While several tools address aspects of this challenge, each has fundamental limitations. REINVENT and similar platforms^26,27^ include built-in diversity filters that penalize structural similarity or recurring scaffolds, but these typically rely on single diversity metrics (*e.g.*, Tanimoto similarity thresholds) with fixed parameters throughout optimization, making them insensitive to the multi-faceted nature of diversity collapse and unable to adapt as chemical space exploration evolves. *Post hoc* benchmarking frameworks such as GuacaMol^32^ and MOSES^37^ provide comprehensive diversity assessment across multiple metrics, enabling systematic comparison of generative models, but operate exclusively after generation is complete and therefore cannot inform real-time intervention or adaptive constraint adjustment. Recent advances in guided generation, including reinforcement learning strategies with shaped rewards^38^ and multi-objective optimization approaches, have improved exploration–exploitation balance, yet these methods lack systematic monitoring capabilities to detect when and how diversity loss occurs during optimization. What remains absent is an integrated framework that combines multi-metric diversity analysis with real-time visualization and adaptive constraint generation, enabling researchers to observe diversity collapse patterns as they emerge and dynamically adjust guidance strategies throughout the molecular discovery process.

The practical consequences of inadequate diversity management became evident in our previous work on singlet fission material discovery with reinforcement-learning-driven generative design workflow.^39^ Despite successful initial optimization using REINVENT4,^27^ the model converged to structurally similar molecules with recurring fragments after 140–150 iterations. Addressing this required 10 manual intervention cycles: analyzing generated molecules, identifying overrepresented fragments, implementing penalties, and restarting optimization. This iterative process, while ultimately successful, highlighted the need for systematic tools that enable real-time diversity monitoring and adaptive constraint implementation throughout the generative process.

This work presents NaviDiv (Navigating Diversity in Chemical Space), a comprehensive framework designed around three core capabilities for chemical diversity management in generative molecular design: comprehensive diversity analysis, interactive visualisation, and adaptive guidance. The framework is built as an interactive web application with a Python backend, designed to monitor and steer the diversity of molecules generated by string-based recurrent neural network (RNN) models. While our implementation is currently tailored for REINVENT4, the approach is generalisable to any string-based model optimized *via* reinforcement learning for the property-directed generation. We demonstrate how these three capabilities work synergistically to enable informed decision-making about diversity–property trade-offs in automated molecular discovery campaigns.

## 2 NaviDiv: a framework for chemical diversity management

NaviDiv is a tool that integrates seamlessly into existing reinforcement learning-based generation pipelines, providing real-time assessment and intervention mechanisms.

### 2.1 Framework overview and design philosophy

**Fig. 1:**
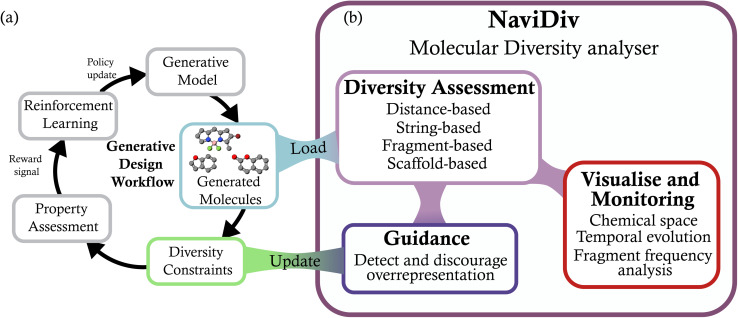
NaviDiv molecular diversity tool. (a) Representation of a generation step with reinforcement learning. (b) Overview of the diversity analysis framework, showcasing the various metrics and visualizations provided to assess chemical diversity during the generative process. The generative model produces molecules that are analyzed using multiple diversity metrics, including representation distance-based, string-based, fragment-based, and scaffold-based approaches. The framework offers visualizations such as 2D chemical space projections and temporal evolution plots to monitor diversity changes over time. The results of the analysis can be used to update a diversity constraints function that can be integrated into the generative model's optimization process.

In a reinforcement-learning-driven generative molecular design workflow (Fig. 1), the model produces candidate molecules at each iteration, which are evaluated using a predefined scoring function that assesses target properties. These scores guide model updates through reinforcement learning, where the model's policy (probability distribution over molecular tokens) is adjusted to favor high-scoring molecules. The optimization process is monitored through two key metrics: the average molecular score, which tracks progress toward the design objective, and the negative log-likelihood under the prior model (the initial generative model), which measures how far the optimized model deviates from its initial training distribution. While higher scores indicate successful optimization, increasing negative log-likelihood signals exploration of novel chemical regions beyond the original training data. However, this optimization process inevitably drives the model toward specific regions of chemical space, potentially missing promising alternatives and reducing overall diversity.

NaviDiv extends this workflow by introducing systematic diversity management capabilities that operate alongside property optimization. Importantly, NaviDiv is designed to be accessible to both computational and experimental chemists, with an intuitive web-based interface that requires no specialized programming knowledge. The framework is built around three core capabilities:

Comprehensive diversity analysis provides multiple complementary metrics to assess chemical diversity from different perspectives, capturing structural, syntactic, and architectural aspects of molecular variation.

Interactive visualisation and monitoring enables real-time observation of diversity evolution through chemical space projections, temporal analysis, and fragment frequency tracking.

Adaptive guidance and constraint generation actively steers the generative process through dynamic penalty functions that maintain desired diversity levels while optimising for target properties.

The framework imposes minimal computational overhead, with performance metrics for each algorithm detailed in the SI. Overall, NaviDiv adds less than 5 seconds per iteration to the optimization workflow, enabling practical integration into routine molecular discovery campaigns without significant performance degradation.

### 2.2 Comprehensive diversity analysis

NaviDiv's diversity analysis capability provides multiple complementary metrics to assess chemical diversity from different perspectives, capturing structural, syntactic, and architectural aspects of molecular variation.

Chemical diversity in this work is defined in broad and context-dependent terms, acknowledging that its meaning varies significantly across different fields.^40,41^ In organic electronics, chemical diversity refers to a wide array of π-conjugated building blocks, which differ in the arrangement and nature of donor-rich and acceptor-rich moieties. Beyond molecular composition, diversity also encompasses molecular symmetry and the potential for ordered spatial arrangement in the solid state, both of which critically influence charge transport, crystallinity, and device performance.^42^ In catalysis, the concept is more constrained: the core catalytic unit often remains unchanged, while diversity is introduced through systematic modifications of the surrounding ligands to optimize activity, selectivity, and stability.^43,44^ In drug discovery, chemical diversity encompasses both structural and functional variation among small molecules, including differences in scaffolds, stereochemistry, and physicochemical properties. This diversity is essential for exploring chemical space and increasing the likelihood of identifying bioactive compounds with novel mechanisms of action.^45,46^

This application-specific nature of chemical diversity motivates our multi-metric approach, where different representations capture complementary aspects of molecular variation. Multiple approaches exist for assessing the chemical diversity of a compound set, each focusing on different molecular features.^47,48^ These methods can be broadly categorized based on the type of representation or structural abstraction they employ. One common strategy is the representation distance-based approach, which uses specific molecular representations—such as structural fingerprints—combined with distance metrics to quantify similarity or dissimilarity between compounds based on their overall structure.^49^ For string-based generative models, we distinguish string-based representations such as simplified molecular input line entry system (SMILES),^50^ where diversity can be evaluated through semantic or syntactic analysis of the molecular encodings, capturing differences in sequence patterns rather than just structural features. Another approach is the scaffold-based method, where molecules are reduced to their core frameworks by algorithmically removing additional functional groups and side chains. These scaffolds are then compared to evaluate diversity at the level of molecular backbones.^51^ A fourth method is the fragment-based approach, in which molecules are systematically broken down into smaller substructures. The diversity is then assessed based on the presence and frequency of these fragments across the dataset.^52^

Building on these established approaches, our tool implements a comprehensive set of metrics specifically designed to assess chemical diversity across multiple representational spaces. These metrics reflect the different approaches discussed above and provide complementary perspectives on molecular variation. The specific methods and their implementation details are described in the SI. The tool also allows users to establish custom diversity metrics based on their specific needs. The implemented metrics include:

#### 2.2.1 Representation distance-based metrics (structural diversity assessment)

Analysis of molecular similarity using fingerprint-based methods (Morgan,^53^ molecular access system (MACCS) keys,^54^ extended connectivity fingerprint (ECFP)^55^) with configurable similarity thresholds. Clustering analysis identifies redundant molecular families and quantifies structural coverage across chemical space.

#### 2.2.2 String-based metrics

Evaluation of SMILES string diversity through *n*-gram analysis, capturing syntactic patterns and sequence-level redundancy. This approach is particularly effective for string-based generative models, as it operates in the same representational space as the generation process.

#### 2.2.3 Fragment-based metrics

Decomposition of molecules into chemically meaningful substructures using established fragmentation algorithms. Frequency analysis of molecular fragments and functional groups provides insights into architectural diversity and identifies over-represented structural motifs.

#### 2.2.4 Scaffold-based metrics

Extraction and comparison of molecular scaffolds to assess core structural diversity. Scaffold frequency analysis highlights dominant frameworks and evaluates the exploration of novel scaffolds over time.

The multi-metric approach ensures comprehensive assessment, as different representations exhibit varying sensitivity to optimization pressure and model collapse phenomena, as demonstrated by the differential degradation patterns observed in the singlet fission campaign.

### 2.3 Interactive visualisation and monitoring

**Fig. 2:**
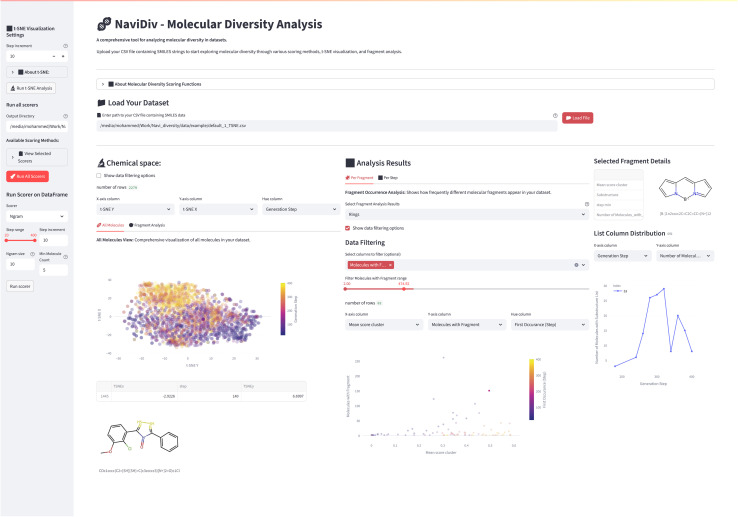
NaviDiv molecular diversity tool. Screenshot of the web application interface showcasing the visualisation capabilities. The dashboard displays multiple diversity metrics, including chemical space projections, temporal evolution plots, and fragment frequency analyses, allowing users to monitor and interpret the diversity of generated molecules in real-time.

NaviDiv's visualisation capability transforms quantitative diversity metrics into interpretable visual representations, enabling researchers to monitor chemical space exploration in real-time. The framework implements multiple visualisation modalities to capture different aspects of molecular diversity and generation dynamics. Fig. 2 shows a screenshot of the web application interface showcasing the visualisation capabilities.

#### 2.3.1 Chemical space projections

Two-dimensional projections of high-dimensional molecular fingerprints (Morgan, MACCS keys, or custom descriptors) using t-distributed stochastic neighbor embedding (t-SNE),^56^ uniform manifold approximation and projection (UMAP),^57^ or principal component analysis (PCA) dimensionality reduction techniques.^58^ These projections reveal clustering patterns, space coverage, and potential over-exploitation of specific chemical regions.

#### 2.3.2 Temporal diversity evolution

Time-series visualisation tracking how diversity metrics evolve throughout the generation process, allowing identification of diversity collapse. Interactive plots enable users to correlate diversity changes with specific reinforcement learning updates or scoring function modifications.

#### 2.3.3 Fragment frequency analysis

Dynamic monitoring of molecular fragment occurrence and frequency shifts over generation steps. Heat maps and frequency distributions highlight emerging structural biases and enable early detection of model collapse toward specific scaffolds.

#### 2.3.4 Multi-metric dashboard

Integrated display combining structural, fingerprint-based, and scaffold diversity metrics in a unified interface. Real-time updates during generation provide immediate feedback on diversity status and trends.

All visualisations are interactive, allowing users to explore specific molecular clusters, investigate outliers, and understand the relationship between diversity patterns and property optimisation objectives. The web-based interface ensures accessibility for both computational and experimental chemists.

### 2.4 Adaptive guidance and constraint generation

The guidance capability actively steers the generative process through dynamic constraint functions that maintain desired diversity levels while optimising for target properties. This capability bridges analysis and action, translating diversity insights into concrete interventions.

#### 2.4.1 Dynamic penalty functions

Real-time generation of penalty terms based on current diversity status and user-defined thresholds. Three complementary constraint algorithms target different diversity aspects (detailed pseudocode in SI Section S2, Algorithms 3–5): similarity-based constraints that penalize structurally redundant molecules *via* Tanimoto clustering; fragment-based constraints that discourage overrepresented molecular substructures; and *n*-gram-based constraints that limit recurring SMILES subsequences, operating directly in the generative model's representational space. Penalties are integrated into the reinforcement learning scoring function.

#### 2.4.2 Multi-metric integration

Combination of penalties from different diversity metrics into a composite constraint function. This holistic approach ensures balanced consideration of structural, syntactic, and architectural diversity aspects.

#### 2.4.3 User-defined thresholds and sensitivity

Customizable parameters allowing users to set acceptable diversity levels and sensitivity of penalties. This flexibility enables tailoring the guidance to specific project goals and chemical spaces.

The guidance system operates in closed-loop with the generative model, continuously updating constraint functions based on real-time analysis results. This ensures responsive adaptation to changing diversity patterns throughout the molecular discovery process, addressing the limitation of the manual iterative approach used in the original singlet fission study, where fragment identification and penalty implementation required 10 separate intervention cycles.

### 2.5 Limitations and future directions

While NaviDiv advances chemical diversity analysis for generative molecular design, several limitations should be acknowledged. Optimal diversity metric selection and threshold determination are context-dependent and require manual tuning, with automated parameter optimization methods remaining an open challenge. Despite being designed as model-agnostic, our implementation focuses on REINVENT4 and has limited validation with other generative architectures or molecular representations. Finally, current metrics may not capture specialized aspects of chemical diversity such as stereochemistry, conformational flexibility, or domain-specific structural motifs. These limitations suggest important directions for future development and indicate areas requiring caution when applying NaviDiv to novel domains or unprecedented scales.

Future developments will focus on extending the framework to additional generative architectures to broaden its applicability beyond string-based RNNs. Incorporating 3D structure-aware diversity metrics that account for conformational flexibility and stereochemistry would further enhance relevance to materials and molecular design applications. Property-aware diversity assessment could integrate functional similarity alongside structural diversity, while multi-objective constraint optimization based on Pareto frontier analysis would enable systematic exploration of diversity–property trade-offs.

## 3 Case study: singlet fission molecular design

To demonstrate NaviDiv's capabilities in a realistic molecular discovery scenario, we apply the framework to singlet fission material design, a challenging optimization problem where maintaining chemical diversity is crucial for discovering novel materials. Singlet fission materials absorb high-energy photons and generate two triplets with half the singlet energy, potentially increasing silicon solar cell efficiency above the Shockley–Queisser limit of 33% up to 45%.^59–61^

This case study showcases all three NaviDiv capabilities: (1) comprehensive diversity analysis across multiple metrics, (2) real-time monitoring of diversity evolution during optimization, and (3) adaptive constraint implementation to preserve diversity while maintaining optimization performance.

Following the same generative design workflow as in our previous work,^39^ we employed REINVENT3.2 to train the generative model on an extended dataset combining FORMED^62^ and GEOM3D,^63^ optimized for organic electronic molecules. Subsequently, REINVENT4 was used for goal-directed generation *via* reinforcement learning, using an evaluation function previously developed to explore the chemical space of molecules with singlet-fission character.^39,62,64^ We specifically use the same evaluation function from our previous work, where we assess the difference in energy between the lowest first singly excited state and the energy of the triplet excited state.^39^ The evaluation function employs ChemProp models^65^ trained on excited state energies from the FORMED dataset to predict these electronic properties. More details about the generative model and the evaluation function are provided in the SI Sections S3 and S5.

Here, we will first show the use of the diversity analyser on a run of the generative model with 1000 iterations of the reinforcement learning step with 100 molecules generated per generation step. Then, we introduce different adaptive constraint functions and assess their impact on the evolution of the different metrics of chemical diversity of the generated molecules.

### 3.1 Evolution of chemical diversity with reinforcement learning steps

**Fig. 3:**
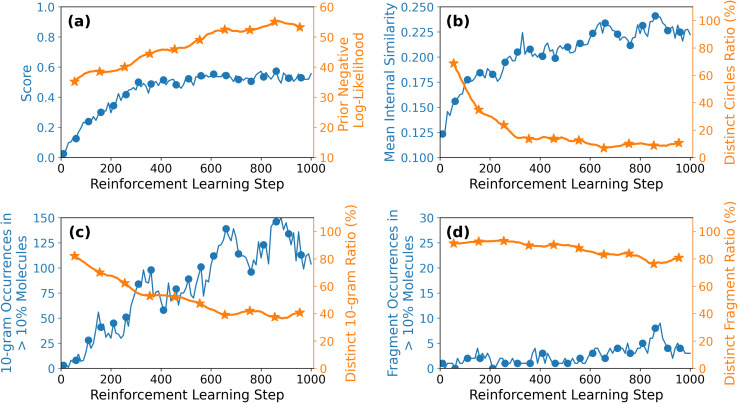
Evolution of molecular optimization and diversity during reinforcement learning. (a) Average molecular score (solid line) and negative log-likelihood under the prior model (dashed line) as a function of reinforcement learning (RL) steps. The increasing score indicates successful optimization, while increased negative log-likelihood reflects exploration of novel chemical space. (b) Structural diversity assessed *via* Morgan fingerprints and Tanimoto similarity. (Left) Mean pairwise similarity among 100 molecules sampled per step. (Right) Number of unique molecular clusters (defined by similarity <0.3). (c) SMILES string diversity analysed using 10-grams. (Left) Fraction of unique 10-grams. (Right) Number of 10-grams present in more than 10% of generated SMILES. (d) Fragment-level diversity measured using chemically relevant substructures identified by a fragmentation algorithm (see SI, Section S1). (Left) Percentage of unique fragments. (Right) Number of fragments occurring in more than 10% of generated molecules. Collectively, these results show increasing optimization at the expense of diversity in some representations, while fragment-level diversity remains relatively robust.

Fig. 3a shows the evolution of the average molecular score alongside the log-likelihood under the original (prior) model throughout the reinforcement learning (RL) process. As training progresses, the average score increases steadily, indicating successful optimization toward the design objective. In contrast, the log-likelihood declines, suggesting that the model increasingly explores regions of chemical space that deviate from the prior distribution. This behavior reflects a deliberate shift toward generating novel and less conventional molecular structures.

Fig. 3b–d track the evolution of molecular diversity across three distinct representations. The detailed implementation of the different metrics is presented in the SI Section S1.

• Fig. 3b: structural diversity is evaluated using Morgan fingerprints with Tanimoto similarity. We examine (i) the mean pairwise similarity among 100 molecules generated per RL step, and (ii) the number of unique molecular clusters, where a cluster is defined as a set of molecules with pairwise similarity greater than 0.3. For reference, a histogram of pairwise similarities in the FORMED database (see Fig. S3) shows that values above 0.2 are rare; hence, a similarity threshold of 0.3 effectively defines structural clusters. Initially, the average similarity is low but it increases substantially during training. The number of unique clusters decreases from nearly 100% (every molecule is in a unique cluster) at the start to approximately 10% after 200 RL steps.

• Fig. 3c: diversity in SMILES string space is analyzed *via* 10-character substrings (10-grams). We report (i) the proportion of unique 10-grams, and (ii) the number of 10-grams occurring in more than 10% of generated SMILES. Initially, nearly all 10-grams are unique, but this fraction decreases to ∼50% by step 200 and ∼40% by step 1000. Concurrently, the number of frequently occurring 10-grams increases from zero to over 100, indicating reduced sequence-level diversity and convergence toward similar SMILES patterns.

• Fig. 3d: fragment-level diversity is assessed based on chemically meaningful substructures derived from a fragmentation algorithm (see SI, Section 1.2). The proportion of distinct fragments drops modestly from 100% to approximately 80% over training. Only a small number (fewer than 10) appear in more than 10% of the generated molecules, suggesting that chemical fragment diversity remains largely preserved, despite convergence in other molecular representations.

Overall, Fig. 3 illustrates the characteristic trade-off between molecular optimization and diversity during reinforcement learning. While the average molecular score increases consistently, this improvement comes at the cost of reduced diversity among the generated molecules. Importantly, the extent of diversity loss varies depending on the representation used: structural similarity based on Morgan fingerprints and sequence-level redundancy measured *via* 10-grams are more substantially impacted than fragment-based metrics. These findings highlight the necessity of employing multiple complementary diversity metrics to fully capture the molecular evolution induced by reinforcement learning. Moreover, this multifaceted perspective becomes essential when designing strategies to constrain model behaviour and preserve chemical diversity. For example, since fragment-based diversity shows only modest degradation (from 100% to 80%) compared to the substantial losses observed in structural similarity and sequence-level metrics, maintaining complete fragment diversity may require stricter constraint thresholds than those needed to preserve sequence-level or fingerprint-based variation, which degrade more rapidly and thus trigger intervention at higher threshold levels.

### 3.2 Impact of diversity constraints

**Fig. 4:**
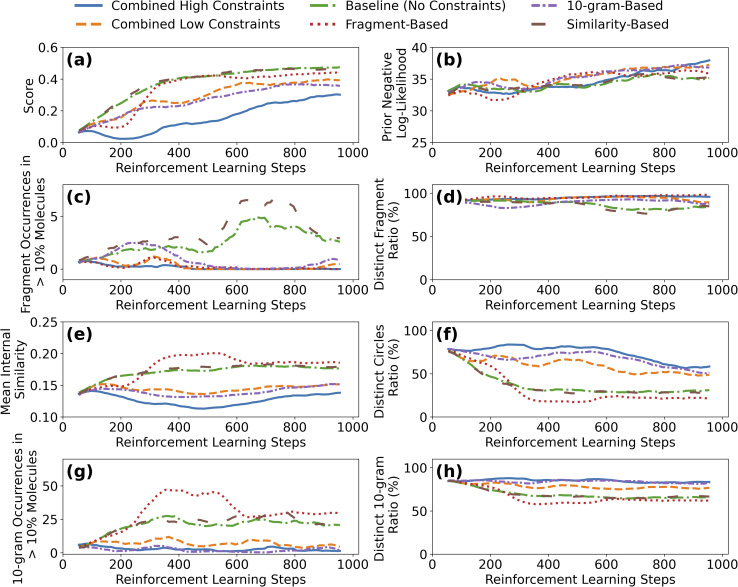
Impact of diversity constraints on molecular optimization and chemical diversity preservation. Evolution of molecular properties and diversity metrics under different constraint regimes across 1000 reinforcement learning steps. Molecular score (a) and prior log-likelihood (b) showing optimization performance *versus* model deviation from initial distribution. Diversity metrics including fragment occurrences in >1% molecules (c) and distinct fragment ratio (d), mean internal similarity using Morgan fingerprints (e) and distinct cluster ratio with similarity threshold 0.3 (f). String-based diversity measured by 10-gram occurrences in >1% molecules (g) and distinct 10-gram ratio (h). Constraint types: no penalty (green), fragment only (red), similarity only (brown), 10-gram only (purple), all weak criteria (orange), and all strong criteria (blue). Results demonstrate that combined constraints (all strong criteria) maintain higher diversity across all metrics while achieving comparable optimization performance, with similarity-based constraints showing particular effectiveness in preserving structural diversity. Here the results are averaged over 5 independent runs of the generative model.

To systematically evaluate the effectiveness of different constraint approaches, we compare six constraint regimes across 1000 reinforcement learning iterations, with each experiment repeated 5 times to ensure statistical significance. This duration is sufficient to observe both the loss of chemical diversity in the unconstrained baseline and the stabilizing effect of each constraint regime, with optimization scores and diversity trends plateauing after approximately 600–800 steps (Fig. 4). This analysis demonstrates how different constraint mechanisms impact both optimization performance and diversity preservation across multiple metrics.

We implement three core constraint types, each targeting different aspects of molecular diversity. Details about the implementation of the diversity constraints is presented in the SI Section S2. Constraint thresholds were established based on the baseline diversity evolution analysis (Fig. 3) and designed to intervene before significant diversity loss occurs. We note that the specific threshold values presented here were selected to demonstrate the framework's capabilities across a range of constraint strictness levels, rather than to represent universally optimal parameters. The optimal thresholds are inherently problem-dependent, varying with the chemical space, generative model, and optimization objective. NaviDiv's real-time monitoring dashboard enables users to observe diversity dynamics under different settings and adapt thresholds to their specific application. Specifically:

#### 3.2.1 Similarity-based constraints

Penalizing structurally similar molecules using Tanimoto similarity and Morgan fingerprints. Here, the molecules are clustered based on pairwise similarity, and any new molecule that falls within the similarity threshold of a cluster is penalized. The molecules to avoid are defined considering the clusters of molecules generated in the previous steps. Clusters exceeding 10 molecules or representing >10% of a generation step trigger penalty application. This threshold corresponds to the point where structural redundancy becomes statistically significant based on the FORMED database distribution.

#### 3.2.2 Fragment-based constraints

Avoiding overrepresented molecular fragments. Fragments appearing in >5% of molecules (minimum 50 total occurrences) are penalized, focusing on fragments >8 non-hydrogen atoms to avoid penalizing ubiquitous small chemical motifs.

#### 3.2.3 N-gram-based constraints

Limiting recurring SMILES subsequences. 10-Character sequences occurring in >3% of molecules (minimum 100 occurrences) are targeted, balancing sequence diversity preservation with practical constraint frequency.

To these three individual constraint types, we add two combined regimes that integrate all three constraints with varying strictness and the baseline case without any constraints:

#### 3.2.4 Combined high constraints (all)

All three constraint types applied with the thresholds defined above.

#### 3.2.5 Combined low constraints (all weak)

All three constraint types applied with relaxed thresholds: 10% and 100 occurrences for fragments, 6% and 200 occurrences for *n*-grams, and 20% and 20 molecules for similarity-based constraints.

#### 3.2.6 Baseline (no constraints)

Standard reinforcement learning without diversity constraints.

The results of the different constraint regimes are shown in Fig. 4. Fig. 4a shows the evolution of the average molecular score, while Fig. 4b shows the evolution of the log-likelihood under the prior model. Regardless of the constraint regime, the average molecular score increases steadily, indicating successful optimization toward the design objective. However, depending on the constraint regime, the score increases at different rates, with the baseline (no constraints) showing the fastest increase. The case with similarity-based constraints shows a similar increase in the average score, while the *n*-gram and combined constraints show a slower increase that does not reach the same level as the baseline even after 1000 steps. The prior negative log-likelihood under the prior model increases for all constraint regimes, indicating that the model increasingly explores regions of chemical space that deviate from the prior distribution. The rate of increase is considerably similar across all constraint regimes for the first 500 steps, but the combined high constraints (all strong criteria) shows a steeper increase in the log-likelihood after 500 steps, indicating that the model is generating molecules that are increasingly different from the prior distribution. This monotonically increasing divergence from the prior across all constraint regimes demonstrates that the model genuinely explores novel regions of chemical space rather than merely exploiting biases of the reward model.

The other panels in Fig. 4 show the evolution of the different diversity metrics with reinforcement learning steps. Compared to the baseline, the fragment-based constraints case does not show any fragment that occurs in more than 10% of the generated molecules, and no change in terms of the unicity of the list of fragments generated with the evolution of the reinforcement learning steps (Fig. 4c and d). Meaning that the fragment-based constraint successfully helps the model generate molecules with very similar fragments. Similarly, the introduction of the *n*-gram constraint function reduces the number of 10-grams that occur in more than 10% of the molecules and maintains the ratio of distinct 10-grams to the same level at the beginning (Fig. 4g and h). On the other hand, the similarity-based constraints case does not show any impact on the evolution of the mean similarity with reinforcement learning steps, as well as a similar reduction in the number of clusters of molecules generated as the baseline (Fig. 4e and f). Even though the approach identifies a large number of molecules to avoid, it does not impact the evolution of the similarity metrics, indicating that the constraint is not effective in this case.

The three diversity-aware constraint regimes (*i.e.*, fragment-based, *n*-gram-based, and similarity-based) show three different impacts on the evolution of the chemical diversity metrics. (1) The introduction of the similarity-based constraints does not preserve the structural diversity of the molecules generated, showing little impact on the evolution of (2) the fragment-based constraints effectively preserve the diversity of the molecular fragments in the generated molecules but do not affect the other diversity metrics, *i.e.*, the mean similarity of the molecules generated or the diversity of the 10-grams. (3) The *n*-gram-based constraints not only preserve the sequence-level diversity but also maintain the diversity across the other metrics, *i.e.*, the mean similarity and the fragment-based diversity. These three cases show that the introduction of diversity-aware constraints can have different impacts on the evolution of the chemical diversity metrics and that having a tool to monitor the chemical diversity of the generated molecules is essential to understand how the constraints impact the chemical space exploration. The *n*-gram based constraints in our case shows the best performance in terms of preserving chemical diversity across all metrics, which is expected as the *n*-gram based constraints are directly applied to the SMILES strings, which are the same representation used by the generative model.

Combining the different constraints seem to overall improve the preservation of the chemical diversity, at the expense of slowing down the rate of increase of the average molecules score. The combined high constraints shows the best performance in terms of preserving chemical diversity across all metrics, with an expected reduction in the rate of increase of the average molecular score. The combined low constraints case shows a slightly worse preservation of the chemical diversity metric as the training progresses, as compared to the combined high constraints, but with a faster rate of increase in the average molecular score. This indicates that the choice of thresholds for the diversity constraints is crucial in balancing the trade-off between optimization performance and chemical diversity preservation. The choice of the thresholds can be adapted to the specific application and the desired level of diversity preservation. Access to a tool that allows monitoring the chemical diversity of the generated molecules is essential to understand how the constraints impact the chemical space exploration and to adapt the thresholds accordingly.

### 3.3 Representational alignment effects

The difference in the effectiveness of constraint types reflects their alignment with the underlying generative model architecture. *n*-Gram constraints operate directly on SMILES sequences—the same representation used by REINVENT4 for token generation—enabling direct influence on the model's sampling probabilities. This representational consistency explains the cross-metric preservation observed: by maintaining string-level diversity, *n*-gram constraints indirectly preserve structural and fragment diversity.

Conversely, similarity-based constraints operate on post-generation molecular fingerprints, creating a representational mismatch with the SMILES-based generation process. While these constraints correctly identify structurally redundant molecules, they cannot effectively guide the character-level sequence generation that determines molecular output. This mismatch explains the limited effectiveness despite accurate structural redundancy detection.

Fragment-based constraints occupy an intermediate position, operating on chemically meaningful substructures that partially align with both SMILES syntax and chemical interpretation. This partial alignment enables targeted effectiveness in preserving fragment diversity while maintaining limited influence on other metrics.

## 4 Conclusion

This work presents NaviDiv, an interactive web-based platform that provides comprehensive tools for monitoring and preserving chemical diversity in generative molecular design, addressing a critical challenge in computational chemistry. Through systematic analysis of multiple diversity metrics and constraint mechanisms applied to singlet fission material discovery, we demonstrate several key findings with broad implications for the field.

Our multi-metric assessment reveals that different diversity measures exhibit varying sensitivity to reinforcement learning optimization, with structural similarity and string-based metrics showing greater vulnerability than fragment-based measures. This differential sensitivity necessitates comprehensive monitoring using complementary representations rather than relying on single metrics.

The representational alignment principle, where constraint effectiveness depends on operating within the same space as the generative model, represents a fundamental design consideration for diversity-aware generative systems. *n*-Gram-based constraints demonstrated superior performance due to direct correspondence with SMILES-based generation, while similarity-based approaches showed limited effectiveness despite correctly identifying structural redundancy. This finding suggests that researchers should prioritize constraint mechanisms compatible with their chosen generative model architectures.

The synergistic benefits of combined constraint strategies, achieving improved diversity preservation compared to individual approaches while maintaining optimization performance within 15% of unconstrained baselines, support multi-objective approaches to generative chemistry. Real-time monitoring capabilities enable dynamic parameter adjustment, addressing limitations of static constraint approaches that become suboptimal as optimization progresses.

The framework's general-purpose design extends beyond the singlet fission case study to any molecular inverse design applications. The integration of analysis, visualization, and constraint generation into an accessible web interface democratizes advanced diversity management tools for both computational and experimental researchers.

By bridging computational power with chemical intuition through systematic diversity management, this work contributes to more effective and interpretable molecular discovery workflows, ultimately supporting accelerated identification of novel compounds with desired properties across diverse application domains from drug discovery to materials science.

## Conflicts of interest

The authors declare no conflicts of interest.

## Supplementary Material

DD-005-D5DD00487J-s001

## Data Availability

The NaviDiv framework, including all source code, documentation, and example datasets, is openly available under an open-source license. The archived version of the software package (version 1.0.0) used in this work is permanently deposited in Zenodo at https://zenodo.org/records/17492074 with DOI: https://doi.org/10.5281/zenodo.17492074. The actively maintained development repository is available on GitHub at https://github.com/LCMD-epfl/NaviDiv, which includes installation instructions, tutorials, and updated features. All molecular structures, diversity analysis results, and reinforcement learning trajectories from the singlet fission case study presented in this work are included in the materials cloud repository https://doi.org/10.24435/materialscloud:jm-x5. The REINVENT4 configurations and evaluation functions used for molecular generation are also provided in the materials cloud repository. Supplementary information (SI): implementation details of all diversity metrics (representation distance-based, fragment-based, scaffold-based, and string-based); pseudocode for the diversity constraint algorithms (similarity-based, fragment-based, and *n*-gram-based); singlet fission evaluation function and machine learning model details; FORMED database statistics including pairwise similarity distributions; detailed experimental setup and REINVENT4 configuration parameters; statistical analysis of constraint regimes with variance across independent runs; a generalizability study applying NaviDiv to QED optimization; and full software implementation details including package architecture. See DOI: https://doi.org/10.1039/d5dd00487j.
