# Supplementary material for: NaviDiv: a web app for monitoring chemical diversity in generative molecular design
Source: Digit Discov. 2026 Mar 30;5(4):1579–89. doi: 10.1039/d5dd00487j (PMC13041631; doi:10.1039/d5dd00487j)
Supplement: DD-005-D5DD00487J-s001 [file DD-005-D5DD00487J-s001.pdf]

Supplementary Information (SI) for Digital Discovery.
This journal is © The Royal Society of Chemistry 2026

Supporting Information for: NaviDiv: A Web App for Monitoring Chemical Diversity in Generative Molecular Design

Mohammed Azzouzi,[*,†] Thanapat Worakul,[†] and Clémence Corminboeuf[†,‡]

†*Laboratory for Computational Molecular Design, Institute of Chemical Sciences and Engineering,*
École Polytechnique Fédérale de Lausanne (EPFL), 1015 Lausanne, Switzerland

‡*National Center for Competence in Research-Catalysis (NCCR-Catalysis),*
École Polytechnique Fédérale de Lausanne (EPFL), 1015 Lausanne, Switzerland

E-mail: Mohammed.azzouzi@epfl.ch

Table of Contents

1 Implementation Details of Diversity Metrics 3

2 Diversity Constraint Algorithms 8

3 Singlet Fission Evaluation Function 10

4 FORMED Database Statistics 12

5 Detailed Experimental Setup 13

6 Statistical Analysis of Constraint Regimes 15

7 Generalizability: QED Optimization Case Study **18**

8 Software Implementation Details **20**

9 Web Application Features **25**

References **35**

1 Implementation Details of Diversity Metrics

The aim of a molecular discovery campaign is to generate a diverse set of molecules with the desired properties, discovering novel molecules or molecular scaffolds that can achieve the desired function. Ensuring diversity in the generated molecules ensures better coverage of the chemical space and reduced chances of over-exploiting specific regions. Expanding the molecules beyond the training chemical space can help improve surrogate models by generating molecules out-of-distribution with high uncertainty related to their properties.

Quantifying diversity in generated molecules can be achieved through different approaches, each focusing on different molecular features. These methods can be broadly categorized based on the type of representation or structural abstraction they employ.

1.1 Representation Distance-Based Metrics

This approach uses molecular representations such as structural fingerprints and distance metrics to quantify similarity or dissimilarity between compounds based on their overall structure.

1.1.1 Morgan Fingerprints and Tanimoto Similarity

Morgan fingerprints are computed using RDKit with the following parameters:

- Radius: 3 (equivalent to ECFP6)
- Number of bits: 2048
- Use features: False
- Use chirality: True

The Tanimoto coefficient between two fingerprints A and B is calculated as:

$$T(A,B) = \frac{|A \cap B|}{|A \cup B|} = \frac{|A \cap B|}{|A| + |B| - |A \cap B|} \tag{1}$$

To evaluate the diversity of a set of generated molecules, we define similarity measures between molecular structures using molecular fingerprints and apply the Tanimoto similarity to quantify pairwise molecular distances. Based on this, we calculate metrics such as the internal diversity (IntDiv), which captures the average dissimilarity within the set.

An alternative approach involves computing the "number of circles" (similar to Renz et al., 2024). In this method, molecules are sequentially selected from the set, and any other molecule within a predefined distance threshold is discarded. This process is repeated until no molecules remain, and the number of selected molecules provides an estimate of diversity. This technique is conceptually related to the leader clustering algorithm.

1.1.2 Clustering Algorithm

Molecular clustering is performed using the following algorithm:

Algorithm 1 Molecular Clustering for Diversity Assessment

Input: Set of molecules M, similarity threshold τ
Output: Number of unique clusters $n_{clusters}$
Initialize empty cluster list $C = \{\}$
for each molecule m_i in M **do**
    $assigned = False$
    for each cluster c_j in C **do**
        if $T(m_i, representative(c_j)) > \tau$ **then**
            Add m_i to cluster c_j
            $assigned = True$
            break
        end if
    end for
    if $assigned == False$ **then**
        Create new cluster with m_i as representative
        Add new cluster to C
    end if
end for
return $|C|$

1.1.3 Dimensionality Reduction and Visualization

For 2D visualization of chemical space, we employ:

- **t-SNE**: t-distributed Stochastic Neighbor Embedding with perplexity = 30, learning rate = 200

1.2 Fragment-Based Metrics Implementation

Chemical diversity can be assessed through analysis of molecular fragments (substructures obtained by systematically decomposing molecules). This approach becomes particularly relevant for larger molecules, where recurring subunits may dominate the chemical space. By collecting and cataloguing fragments present across a dataset, we can evaluate their frequency of occurrence and identify overrepresented motifs.

1.2.1 Fragmentation Algorithm

Molecular fragmentation is performed using the following implementation based on the rdScaffoldNetwork implementation in RDKit:[1]

Algorithm 2 Fragmentation Algorithm

Input: Input molecule mol, minimum number of atoms threshold min_num_atoms
Output: List of fragment SMILES strings $fragments$
Initialize scaffold network parameters
$fragments \leftarrow \{MolToSmiles(mol)\}$ ▷ Initialize with original molecule
$net \leftarrow buildScaffoldNetwork([mol], params)$
for each $scaffold$ in $net.nodes$ **do**
    $scaffold\_mol \leftarrow MolFromSmiles(scaffold)$
    if $scaffold\_mol.GetNumAtoms() \geq min\_num\_atoms$ **then**
        $fragments.add(scaffold)$
    end if
    $removed\_frags \leftarrow removeSubstructure(mol, scaffold)$
    for each $frag$ in $removed\_frags$ **do**
        if $frag.GetNumAtoms() \geq min\_num\_atoms$ **then**
            $fragments.add(MolToSmiles(frag))$
        end if
    end for
end for
Filter out invalid SMILES:
$fragments \leftarrow \{smi \in fragments : MolFromSmiles(smi) \neq null\}$
return $fragments$ as list

1.2.2 Fragment Abstraction Levels

Molecular fragments can be compared through simplified representations that abstract away detailed chemical information. We implement three levels of fragment abstraction:

1. **Full**: Complete chemical structure with all atom and bond information

2. **Wireframe**: Bond orders removed, only connectivity preserved

3. **Elemental**: All atoms replaced with carbon, only topology preserved

These abstraction techniques allow for more generalized comparison of molecular patterns, particularly useful when focusing on topological or connectivity-based features rather than specific chemical identities.

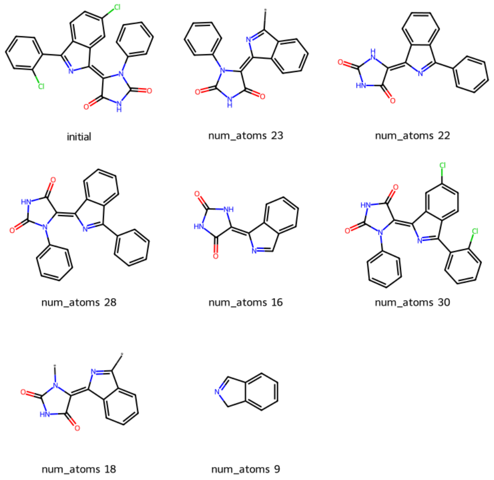

Figure S1: Example of molecular fragmentation algorithm applied to a molecule. Fragments are limited to those with at least 8 non-hydrogen atoms.

1.3 Scaffold-Based Metrics Implementation

Scaffold-based diversity analysis focuses on the core structural frameworks of molecules, distinguishing it from fragment-based approaches that examine smaller, often disconnected substructures. Molecules are reduced to their scaffolds, typically defined as central ring systems and linkers after removing peripheral substituents.

1.3.1 Scaffold Extraction

We implement multiple scaffold definitions to capture different aspects of molecular architecture:

1. **Bemis-Murcko Scaffolds**: Using RDKit's `GetScaffoldForMol` to extract bioactive cores
2. **Generic Scaffolds**: Atom types replaced with generic atoms to focus on topology
3. **Ring System Scaffolds**: Only ring systems preserved, removing linkers
4. **GNN-derived Scaffolds**: Scaffolds extracted from model embeddings where atom contributions to properties are considered

The definition of scaffold can be tailored to specific applications. For example, in drug discovery, Bemis-Murcko scaffolds capture bioactive cores, while in materials science, scaffolds may be defined based on electronic or steric properties.

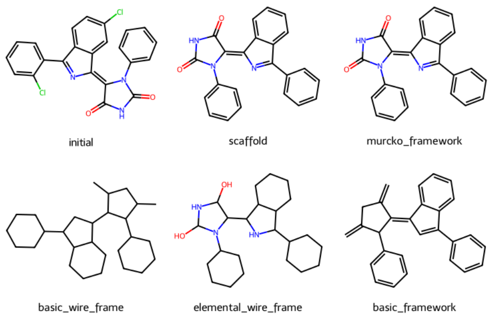

Figure S2: Example of extracting different types of molecular scaffolds from the same molecule, showing the various levels of structural abstraction.

1.4 String-Based Metrics Implementation

Since the generative models used are language-based—typically relying on SMILES (Simplified Molecular Input Line Entry System) representations—we can quantify molecular diversity through string-based metrics. This approach analyzes the frequency of recurring substrings, commonly referred to as n-grams.

1.4.1 N-gram Analysis

An n-gram refers to a contiguous sequence of n characters or tokens in SMILES strings. By examining how often specific n-grams occur across generated molecules, we can assess redundancy or variation in the output. High frequency of certain n-grams indicates limited diversity and over-representation of particular structural motifs, while uniform distribution suggests broader exploration of chemical space.

1.4.2 Statistical Measures

Beyond frequency analysis, n-gram-based metrics can be extended to calculate:

- **Entropy**: Reflects unpredictability and richness of the generated chemical language
- **Distribution comparison**: Compare n-gram distributions in generated molecules to reference datasets (ChEMBL, ZINC)
- **Diversity coefficients**: Measure how well the model replicates or diverges from known chemical diversity

Because language-based models encode chemistry as text, these methods serve as natural analogs to traditional structure-based diversity metrics, offering scalable and interpretable means of assessing diversity at the representation level.

2 Diversity Constraint Algorithms

A promising approach to promoting chemical diversity involves penalizing molecules when they exhibit excessive similarity, share common backbones, or contain overrepresented molecular fragments. These penalties are directly tied to desired chemical diversity and can be quantified through penalty scores.

Defining these penalties within the fitness function requires thorough analysis of generated molecules. Based on findings, users can adjust penalization functions accordingly. This

section provides detailed algorithmic procedures for the diversity-aware constraint functions mentioned in the main text.

2.1 Similarity-Based Constraints

Algorithm 3 Similarity-Based Diversity Constraint

```
Input: Generated molecules M_new, molecules to avoid M_avoid, threshold τ_sim
Output: Penalized scores for M_new
for each molecule m_i in M_new do
    penalty = 1.0                                  ▷ Default score multiplier
    for each molecule m_j in M_avoid do
        if T(m_i, m_j) > τ_sim then
            penalty = 0.0                          ▷ Complete penalty
            break
        end if
    end for
    Apply penalty to score of m_i
end for
Update M_avoid with cluster representatives from M_new if cluster size > threshold
```

2.2 Fragment-Based Constraints

Algorithm 4 Fragment-Based Diversity Constraint

```
Input: Generated molecules M_new, fragment frequency dict F_freq, threshold τ_frag
Output: Penalized scores for M_new
for each molecule m_i in M_new do
    F_i = fragment(m_i)                            ▷ Extract fragments
    penalty = 1.0
    for each fragment f in F_i do
        if F_freq[f] > τ_frag then
            penalty = 0.0
            break
        end if
    end for
    Apply penalty to score of m_i
    Update F_freq with fragments from F_i
end for
```

2.3 N-Gram-Based Constraints

Algorithm 5 N-Gram-Based Diversity Constraint

Input: Generated SMILES S_{new}, n-gram frequency dict N_{freq}, n-gram size n, threshold τ_{ngram}
Output: Penalized scores for molecules
for each SMILES s_i in S_{new} **do**
    $N_i = extract\_ngrams(s_i, n)$ ▷ Extract n-grams
    $penalty = 1.0$
    for each n-gram g in N_i **do**
        if $N_{freq}[g] > \tau_{ngram}$ **then**
            $penalty = 0.0$
            break
        end if
    end for
    Apply penalty to score of molecule from s_i
    Update N_{freq} with n-grams from N_i
end for

3 Singlet Fission Evaluation Function

This section details the singlet fission evaluation function used in the case study.

3.1 Energy-Based Scoring

The singlet fission evaluation function follows the energy-based scoring methodology developed in our previous work.[2,3] Briefly, viable SF chromophores must satisfy three vertical excited-state criteria: near-thermoneutral singlet fission driving energy, emission in the absorption range of silicon , and absorption of abundant photons in the solar spectrum. The energy score function quantifies molecular suitability by computing the minimum signed distance to the optimal energy region defined by the triangular coordinates (1.5 eV, 3.8 eV), (1.9 eV, 3.8 eV), and (1.5 eV, 3.0 eV) in space. Complete mathematical formulation and implementation details are provided in the referenced prior work.

3.2 Machine Learning Model Details

To predict the excited-state properties of interest, a GNN-based multi-target property prediction model was trained on the FORMED database using Chemprop v1.5.2. The Chemprop model architecture consists of a 3-layer GNN with a hidden size of 300 and a dropout probability of 0.2. The dataset was randomly split into training, validation, and test sets with an 80/10/10 ratio. More details about the model can be found in our previous work.[3]

We note that the ChemProp model serves exclusively as a fixed scoring function during the reinforcement learning optimization and is not further fine-tuned or evaluated on its test set in this work. The generative model (REINVENT4) is trained on a larger combined dataset (FORMED + GEOM3D). Although molecular overlap may exist between the ChemProp test split and the generative training data, this does not affect the conclusions of this study, which focus on diversity monitoring and constraint behavior rather than on benchmarking property prediction accuracy.

3.3 Additional Molecular Filters

The evaluation function includes additional sorting functions to filter out molecules that do not meet specific criteria:

- Molecular weight: 300-800 Da
- Synthetic accessibility score: ≤ 3

3.4 Data Attrition Rates

Table S1 summarizes the percentage of molecules filtered at each processing stage across all constraint configurations. Invalid SMILES strings are discarded entirely as they cannot be parsed into molecular structures. Molecules with molecular weight outside the 300–800 Da

range or with a synthetic accessibility score above the threshold are not removed from the generated pool; instead, they receive a score of 0 and are still included in all diversity analyses. This ensures that the diversity metrics reflect the full distribution of valid molecules produced by the generative model, while the scoring function penalizes undesirable candidates.

Table S1: Percentage of molecules affected at each processing stage, averaged across experiments (mean ± std). MW: molecular weight range 300–800 Da; SA: synthetic accessibility score (transformed score < 0.5). Invalid SMILES are discarded; molecules failing MW or SA criteria receive a score of 0 but remain in the diversity analysis.

Configuration	% Invalid SMILES	% MW outside range	% SA penalized
Combined High	6.1 ± 0.2	25.1 ± 1.4	4.5 ± 0.6
Combined Low	5.0 ± 0.2	17.1 ± 1.2	1.7 ± 0.2
Baseline	5.0 ± 0.2	8.3 ± 0.4	1.0 ± 0.0
Fragment-Based	4.1 ± 0.2	10.8 ± 0.8	1.3 ± 0.2
10-gram-Based	6.4 ± 0.4	18.2 ± 0.5	2.2 ± 0.2
Similarity-Based	4.9 ± 0.3	8.0 ± 0.6	1.0 ± 0.1
Overall	5.2 ± 0.8	13.8 ± 6.3	1.9 ± 1.2

4 FORMED Database Statistics

This section provides statistical analysis of the FORMED database used for model training.

4.1 Pairwise Similarity Distribution

Figure S3 shows the distribution of pairwise Tanimoto similarities in the FORMED database. The analysis reveals:

- Mean similarity: 0.15 ± 0.08
- Median similarity: 0.12
- 95th percentile: 0.32
- Pairs with similarity > 0.3: 2.1%

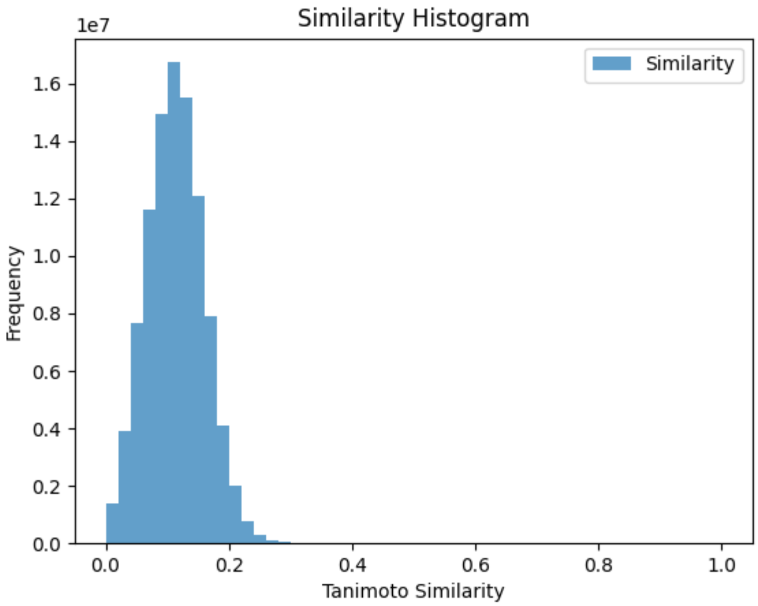

Figure S3: Distribution of pairwise Tanimoto similarities in the FORMED database using Morgan fingerprints. The low average similarity indicates high structural diversity in the training set.

4.2 Fragment and Scaffold Analysis

Statistical analysis of fragments and scaffolds in FORMED:

- Total unique fragments (size $\geq$ 8 atoms): 45,672
- Most common fragment frequency: 0.8% of molecules
- Total unique Bemis-Murcko scaffolds: 12,334
- Scaffold coverage: 89% of molecules have unique scaffolds

5 Detailed Experimental Setup

5.1 Problem Definition and Generative Model

In this work, we revisit a problem previously explored related to singlet fission molecules, focusing on energy requirements related to the difference in energy between the first singlet

and first triplet states. We use a pre-trained graph neural network for property prediction.

We employ REINVENT4 trained on the GEOM3D and FORMED datasets, providing a diverse foundation for organic electronic molecules with an updated vocabulary suitable for the target application.

5.2 REINVENT4 Configuration

The reinforcement learning setup uses the following parameters:

- **Prior model**: REINVENT4 trained on FORMED + GEOM3D
- **Agent update frequency**: Every 10 steps
- **Batch size**: 100 molecules per step
- **Learning rate**: 0.0001
- **Sigma**: 120 (DAP regularization parameter)
- **Total steps**: 1000

The DAP (Difference between Augmented and Posterior) strategy is included to enhance exploration capabilities, though as shown in the results, additional diversity constraints are necessary to maintain chemical diversity after extended reinforcement learning.

5.3 Diversity Constraint Thresholds

Table S2 summarizes the threshold parameters used for different constraint regimes.

Table S2: Threshold parameters for diversity constraint regimes

Constraint Type	Metric	Strong	Weak
Similarity-based	Cluster size (%)	10	20
	Cluster size (abs.)	10	20
	Similarity threshold	0.3	0.3
Fragment-based	Fragment freq. (%)	5	10
	Fragment freq. (abs.)	50	100
	Min. fragment size	8	8
N-gram-based	N-gram freq. (%)	3	6
	N-gram freq. (abs.)	100	200
	N-gram size	10	10

6 Statistical Analysis of Constraint Regimes

6.1 Variance Across Independent Runs

Figure S4 presents comprehensive variance analysis showing mean ± standard deviation across five independent runs for all six constraint regimes (Combined High Constraints, Combined Low Constraints, Baseline, Fragment-Based, 10-gram-Based, and Similarity-Based) and all eight diversity metrics presented in the main text Figure 4.

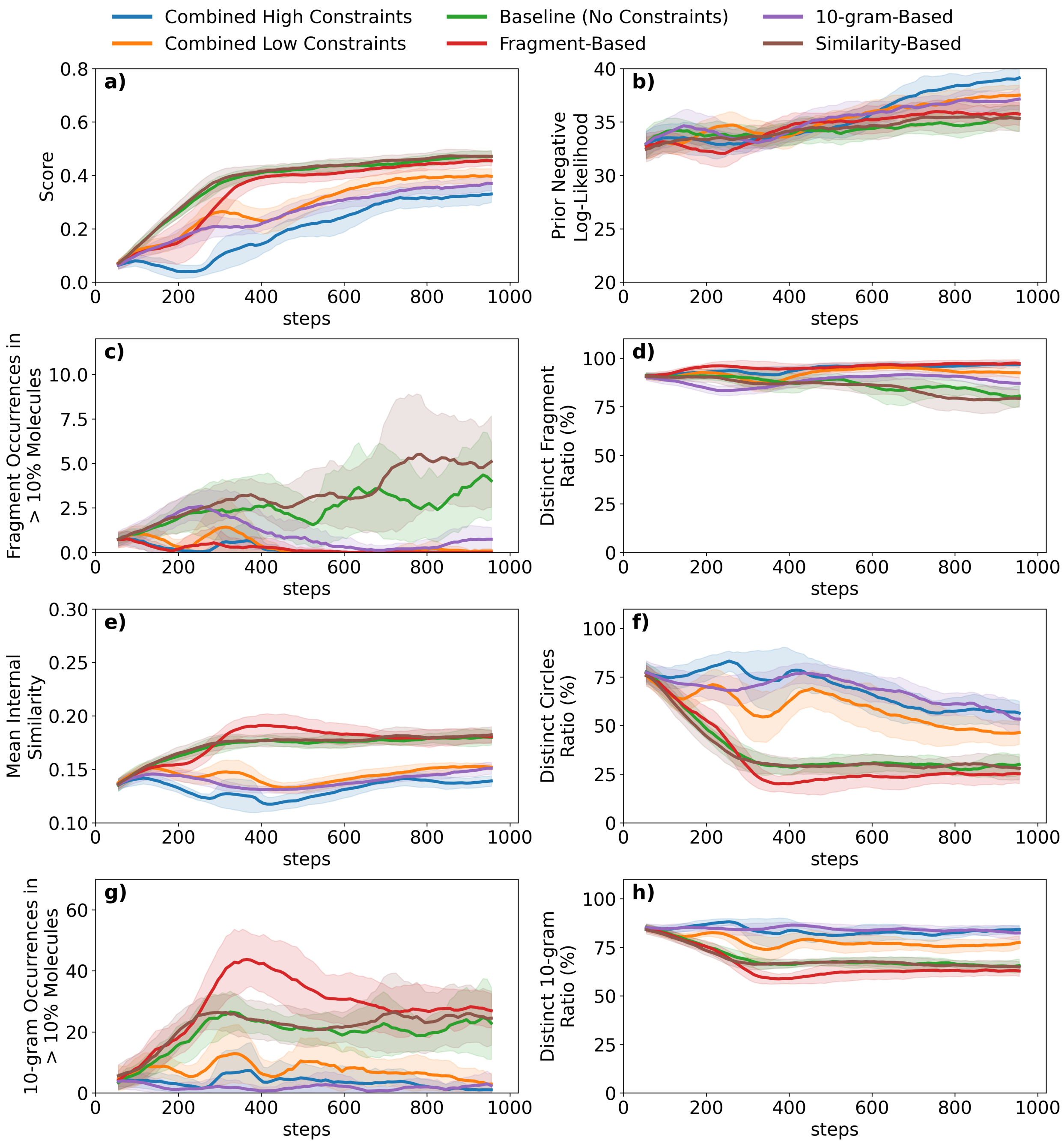

Figure S4: **Variance analysis across constraint regimes.** Evolution of diversity metrics showing mean (solid lines) and standard deviation (shaded regions) across five independent runs for all constraint configurations: (a) molecular score, (b) prior negative log-likelihood, (c) fragment occurrences in >10% of molecules, (d) distinct fragment ratio, (e) mean internal similarity, (f) distinct cluster ratio, (g) 10-gram occurrences in >10% of molecules, (h) distinct 10-gram ratio. The shaded regions demonstrate the statistical robustness of the trends reported in the main text.

6.2 Numerical Summary Statistics

Table S3 provides quantitative summaries (mean ± standard deviation) for key diversity and optimization metrics at representative time points throughout the reinforcement learning process.

Table S3: Mean ± standard deviation of diversity metrics across 5 runs at selected RL steps.

Method	Step	Score	Prior NLL	Frag. Occ. (>10%)	Distinct Frag. (%)	Mean Int. Sim.	Distinct Circles (%)	10-gram Occ. (>10%)	Distinct 10-gram (%)
Combined High	250	0.038 ± 0.015	32.1 ± 1.2	0.000 ± 0.000	93.8 ± 1.1	0.124 ± 0.006	83.3 ± 4.0	1.60 ± 1.52	88.3 ± 1.1
	500	0.222 ± 0.031	34.4 ± 0.6	0.000 ± 0.000	96.4 ± 1.3	0.123 ± 0.008	72.5 ± 10.7	1.20 ± 2.17	81.4 ± 5.0
	750	0.322 ± 0.028	37.9 ± 1.6	0.000 ± 0.000	94.8 ± 1.9	0.141 ± 0.006	55.7 ± 5.5	4.00 ± 3.74	81.7 ± 3.9
	1000	0.347 ± 0.037	40.2 ± 0.5	0.000 ± 0.000	96.7 ± 1.4	0.142 ± 0.008	52.7 ± 7.4	1.00 ± 1.41	83.5 ± 2.9
Combined Low	250	0.231 ± 0.051	34.7 ± 1.0	0.400 ± 0.894	90.4 ± 1.2	0.142 ± 0.005	67.1 ± 10.1	6.80 ± 3.77	81.6 ± 3.2
	500	0.301 ± 0.034	35.9 ± 0.5	0.000 ± 0.000	95.0 ± 1.3	0.134 ± 0.006	68.8 ± 10.9	14.6 ± 7.0	77.0 ± 4.6
	750	0.408 ± 0.035	36.5 ± 1.6	0.000 ± 0.000	94.3 ± 1.8	0.146 ± 0.006	52.4 ± 11.9	8.80 ± 6.87	75.2 ± 2.0
	1000	0.395 ± 0.037	38.2 ± 0.9	0.000 ± 0.000	91.6 ± 3.2	0.152 ± 0.004	45.7 ± 7.8	5.00 ± 6.40	77.2 ± 2.7
Baseline	250	0.318 ± 0.039	34.6 ± 1.2	2.50 ± 0.58	90.0 ± 2.2	0.167 ± 0.005	39.3 ± 3.9	18.5 ± 4.8	73.0 ± 2.2
	500	0.438 ± 0.022	33.9 ± 0.8	1.00 ± 0.82	90.0 ± 1.4	0.172 ± 0.003	30.5 ± 6.1	18.0 ± 1.4	67.8 ± 2.2
	750	0.447 ± 0.013	34.6 ± 0.9	1.75 ± 0.96	87.3 ± 4.0	0.174 ± 0.003	30.0 ± 6.7	21.0 ± 5.9	68.1 ± 1.0
	1000	0.484 ± 0.024	34.9 ± 1.1	3.75 ± 1.26	80.6 ± 4.6	0.178 ± 0.005	29.9 ± 3.3	18.5 ± 6.1	67.0 ± 2.7
Fragment-Based	250	0.208 ± 0.085	32.3 ± 1.1	0.800 ± 1.095	95.7 ± 3.7	0.157 ± 0.009	43.8 ± 8.5	23.2 ± 7.9	71.2 ± 2.9
	500	0.398 ± 0.027	35.2 ± 0.3	0.200 ± 0.447	94.0 ± 2.5	0.190 ± 0.016	24.2 ± 6.2	39.2 ± 12.7	61.2 ± 5.1
	750	0.426 ± 0.033	35.2 ± 1.0	0.000 ± 0.000	97.9 ± 1.2	0.182 ± 0.005	20.9 ± 2.4	26.4 ± 9.4	63.1 ± 2.5
	1000	0.445 ± 0.023	35.8 ± 1.6	0.000 ± 0.000	96.0 ± 3.6	0.179 ± 0.005	25.0 ± 5.8	24.2 ± 2.9	61.6 ± 2.8
10-gram-Based	250	0.198 ± 0.039	33.9 ± 2.1	3.20 ± 0.84	82.4 ± 1.0	0.143 ± 0.006	66.7 ± 7.2	2.60 ± 3.44	84.2 ± 2.4
	500	0.288 ± 0.031	35.9 ± 0.9	0.200 ± 0.447	92.0 ± 1.3	0.131 ± 0.003	76.0 ± 4.8	2.80 ± 2.95	84.5 ± 3.4
	750	0.356 ± 0.012	35.7 ± 0.7	0.600 ± 0.548	90.6 ± 1.4	0.143 ± 0.001	61.0 ± 6.8	0.400 ± 0.548	83.8 ± 2.2
	1000	0.350 ± 0.048	37.7 ± 1.3	1.00 ± 0.71	86.7 ± 2.1	0.152 ± 0.005	53.0 ± 4.6	0.800 ± 1.095	82.6 ± 3.4
Similarity-Based	250	0.323 ± 0.044	33.1 ± 1.0	2.20 ± 0.84	91.0 ± 1.8	0.171 ± 0.002	36.6 ± 3.9	25.2 ± 1.8	69.3 ± 1.7
	500	0.428 ± 0.018	34.3 ± 1.2	2.60 ± 1.52	87.7 ± 1.0	0.177 ± 0.005	27.2 ± 4.3	21.4 ± 3.3	66.7 ± 1.8
	750	0.450 ± 0.020	35.6 ± 1.2	5.80 ± 3.03	79.7 ± 7.4	0.182 ± 0.008	28.3 ± 8.1	26.6 ± 16.5	65.4 ± 3.9
	1000	0.472 ± 0.028	35.1 ± 1.3	6.40 ± 3.97	77.6 ± 5.6	0.185 ± 0.009	26.7 ± 5.3	24.6 ± 8.6	63.9 ± 2.9

7 Generalizability: QED Optimization Case Study

To demonstrate the generalizability of NaviDiv beyond the singlet fission application presented in the main text, we apply the framework to a widely used benchmark objective: the Quantitative Estimate of Drug-likeness (QED). QED is a standard scoring function in drug discovery that evaluates molecular desirability based on multiple physicochemical properties including molecular weight, logP, number of hydrogen bond donors and acceptors, polar surface area, number of rotatable bonds, and the presence of undesirable structural features. Unlike the singlet fission evaluation function, which relies on machine learning models trained on quantum chemical data, QED is an interpretable, rule-based metric, making it complementary for assessing the framework's applicability across different optimization objectives.

We employ the same experimental setup as described in the main text: REINVENT4 with 1000 reinforcement learning steps, 100 molecules generated per step, and the same six constraint regimes (Baseline, Fragment-Based, Similarity-Based, 10-gram-Based, Combined Low Constraints, and Combined High Constraints). All results are averaged over 5 independent runs.

Figure S5 presents the evolution of the QED score and key diversity metrics across all constraint regimes. The QED optimization exhibits several notable differences from the singlet fission case study. The baseline QED scores (Figure S5a) start higher and converge more rapidly, reflecting the relatively easier optimization landscape compared to the multi-objective singlet fission function. Despite these differences, the diversity dynamics follow similar qualitative patterns: unconstrained optimization leads to progressive diversity loss, while the combined constraint regimes maintain higher diversity across fragment-based (Figure S5c), structural similarity (Figure S5d), and string-based (Figure S5e) metrics.

These results confirm that the diversity monitoring and constraint mechanisms implemented in NaviDiv generalize across different optimization objectives and chemical spaces, supporting the framework's applicability as a domain-agnostic tool for managing chemical diversity in

generative molecular design.

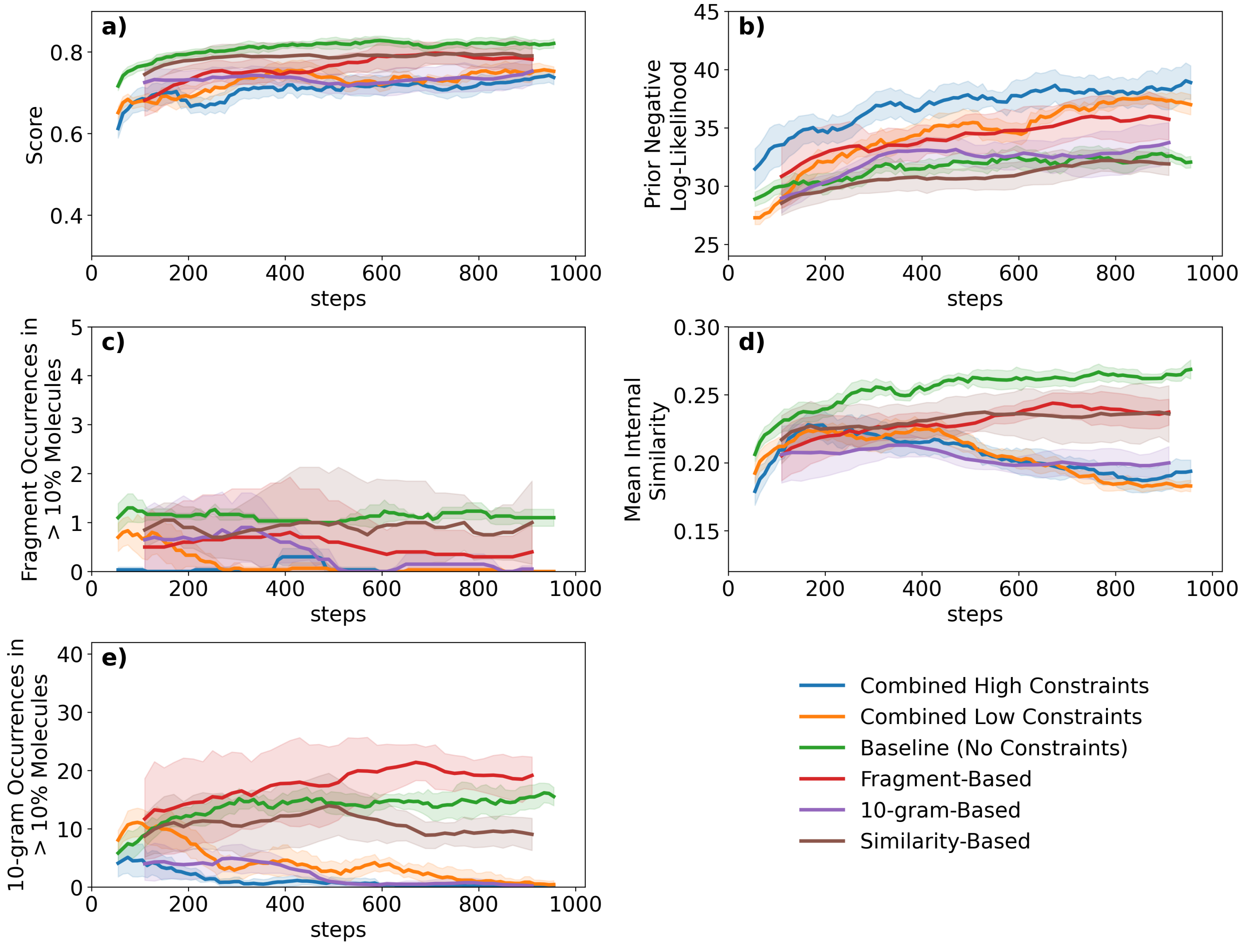

Figure S5: **QED optimization: diversity metrics across constraint regimes.** Evolution of (a) QED score, (b) prior negative log-likelihood, (c) fragment occurrences in >10% of molecules, (d) mean internal similarity, and (e) 10-gram occurrences in >10% of molecules across 1000 reinforcement learning steps. Solid lines represent mean values and shaded regions represent standard deviation across five independent runs. Constraint regimes: Combined High Constraints (blue), Combined Low Constraints (orange), Baseline (green), Fragment-Based (red), 10-gram-Based (light purple), and Similarity-Based (dark purple). The results demonstrate that NaviDiv's diversity monitoring and constraint mechanisms generalize to standard drug discovery objectives.

8 Software Implementation Details

8.1 Architecture Overview

NaviDiv is implemented as a modular Python framework with a **Streamlit web interface** that provides an intuitive platform for molecular diversity analysis. The application follows a three-tier architecture designed for scalability, modularity, and ease of use.

8.1.1 Core Analysis Engine

- **Base Scorer Framework**: Abstract `BaseScore` class providing standardized interface for all diversity metrics
- **Modular Scorer System**: Independent scorer modules for different diversity aspects
- **Efficient Data Processing**: Optimized pandas/numpy operations with RDKit integration
- **Session Management**: Streamlit session state for maintaining analysis context

8.1.2 Web Interface Layer

- **Responsive UI**: Built with Streamlit for cross-platform compatibility
- **Interactive Visualizations**: Plotly-powered 2D/3D molecular space projections
- **Real-time Analysis**: Live updates during diversity scoring operations
- **File Management**: Support for CSV upload, file browsing, and example datasets

8.1.3 Analysis Modules

- **Fragment Analysis**: BRICS fragmentation and frequency-based scoring
- **Scaffold Analysis**: Murcko and custom scaffold generation with GNN models
- **Similarity Clustering**: Fingerprint-based molecular clustering with multiple metrics

- **String Analysis**: N-gram SMILES pattern recognition
- **Diversity Metrics**: Comprehensive statistical diversity measurements

8.2 Core Scoring Framework Implementation

8.2.1 BaseScore Architecture

The foundation of NaviDiv's scoring system is the abstract `BaseScore` class, which provides a standardized interface for all diversity metrics:

Listing 1: BaseScore Framework Structure

```
class BaseScore:
    """Abstract base class implementing common scoring functionality"""

    def get_count(self, smiles_list: list[str]) -> tuple[pd.DataFrame, None]:
        """Count occurrences of fragments/patterns in dataset"""

    def add_score_metrics(self, smiles_list: list[str],
                          scores: list[float]) -> pd.DataFrame:
        """Calculate score correlations and statistical metrics"""

    def _comparison_function(self, smiles: str, fragment: str) -> bool:
        """Abstract method for fragment presence detection"""

    def additional_metrics(self) -> dict[str, Any]:
        """Abstract method for scorer-specific metrics"""
```

Key Features:

- **Standardized Interface**: All scorers inherit common methods for consistency

- **Parallel Processing**: Fragment analysis parallelized using process pools
- **Memory Optimization**: Efficient data structures for large molecular datasets
- **Statistical Robustness**: Median-based scoring to handle outliers

8.2.2 Molecular Preprocessing Pipeline

The preprocessing pipeline ensures consistent molecular representation across all analyses:

1. **SMILES Validation**: RDKit-based molecular parsing with comprehensive error handling
2. **Canonical Representation**: Standardized SMILES generation for consistency
3. **Fragment Generation**: Multiple fragmentation approaches (BRICS, custom patterns)
4. **Fingerprint Calculation**: Morgan fingerprints with configurable parameters

8.3 Diversity Scoring Implementations

8.3.1 Fragment-Based Analysis (`FragmentScorer`)

Algorithm Implementation:

- **BRICS Fragmentation**: Breaks molecules at chemically meaningful bonds using RDKit's BRICS implementation
- **Fused Ring Handling**: Special processing for complex ring systems to prevent over-fragmentation
- **Frequency Analysis**: Statistical analysis of fragment occurrence patterns with configurable thresholds
- **Score Correlation**: Identifies fragments correlating with activity scores using median-based statistics

Performance: Approximately 2-3 seconds per 100 molecules on standard CPU hardware.

Listing 2: Fragment Analysis Core Algorithm

```
def get_fragments(self, smiles_list: list[str]) -> list[str]:
    """Extract fragments using BRICS and fused ring fragmentation"""
    fragments = []
    for smiles in smiles_list:
        mol = Chem.MolFromSmiles(smiles)
        if mol:
            # BRICS fragmentation
            brics_frags = BRICS.BRICSDecompose(mol)
            # Custom fused ring handling
            ring_frags = self._get_fused_rings(mol)
            fragments.extend(list(brics_frags) + ring_frags)
    return fragments
```

8.3.2 Scaffold Analysis (`Scaffold_scorer`)

Multiple Scaffold Types Supported:

- **Murcko Scaffolds**: Classical ring system + linker approach using RDKit implementation
- **CSK Scaffolds**: Carbon skeleton representations for structural diversity
- **GNN Scaffolds**: Machine learning-based scaffold identification using graph neural networks
- **Custom Frameworks**: User-definable scaffold patterns with flexible matching criteria

Implementation Details:

- **Canonical Scaffold Generation**: Ensures consistent scaffold representations across analyses

- **Hierarchical Analysis**: Multi-level scaffold decomposition for comprehensive structural assessment
- **Activity Correlation**: Links scaffold presence to molecular property values

Listing 3: Scaffold Generation Algorithm

```
def get_scaffold(self, smiles: str) -> Chem.Mol:
    """Generate molecular scaffold based on configured type"""
    mol = Chem.MolFromSmiles(smiles)
    if not mol:
        return None

    if self.scaffold_type == "murcko":
        return MurckoScaffold.GetScaffoldForMol(mol)
    elif self.scaffold_type == "csk":
        return self._get_carbon_skeleton(mol)
    elif self.scaffold_type == "gnn":
        return self._get_gnn_scaffold(mol)
```

8.3.3 Molecular Clustering (`ClusterSimScorer`)

Similarity Metrics:

- **Tanimoto Coefficient**: Primary similarity measure for molecular fingerprints
- **Dice Coefficient**: Alternative similarity metric for comparative analysis
- **Euclidean Distance**: Continuous space similarity measurements for clustering

Clustering Algorithms:

- **Hierarchical Clustering**: Agglomerative clustering with configurable linkage criteria

- **HDBSCAN**: Density-based clustering for identifying irregular cluster shapes
- **Threshold Clustering**: Simple distance-based grouping with user-defined cutoffs

8.3.4 String-Based Analysis (`NgramScorer`)

N-gram Analysis Implementation:

- **Configurable N**: Variable-length substring analysis (typically 3-6 characters)
- **SMILES Patterns**: Identifies recurring chemical motifs as string patterns
- **Levenshtein Distance**: Quantifies molecular string similarity using edit distance
- **Pattern Frequency**: Statistical analysis of motif occurrence with significance testing

9 Web Application Features

9.0.1 File Management System

The application provides multiple modalities for data input, designed for flexibility and ease of use:

Listing 4: Multi-Modal Data Loading Implementation

```
def load_file_section() -> str:
    """Handle file loading with expandable interface"""
    # Check if file already loaded
    file_loaded = (hasattr(st.session_state, "file_path") and
                   st.session_state.file_path)

    # Expandable interface that collapses when file is loaded
    with st.expander("Load Your Dataset", expanded=not file_loaded):
        # Tab-based interface for different loading methods
```

```
tab_upload, tab_path = st.tabs(["Upload File", "File Path"])

# CSV upload, file browsing, example selection
# Automatic validation and format checking
```

Features:

- **Drag-and-Drop Upload**: Browser-based file selection with automatic validation
- **Project File Browser**: Automatic discovery of example datasets in project directories
- **Path Validation**: Real-time file existence and format checking
- **Session Persistence**: Maintains file state across user interactions

9.0.2 Interactive Visualization System

Chemical Space Projection:

- **t-SNE Implementation**: Scikit-learn based dimensionality reduction with optimized parameters
- **Real-time Rendering**: Plotly-based interactive scatter plots with zoom and pan capabilities
- **Molecular Highlighting**: Click-to-highlight specific compounds with structural information
- **Evolution Tracking**: Temporal analysis of diversity changes across generation steps

Performance Optimizations:

- **Incremental Updates**: Only recompute analyses when input data changes
- **Caching System**: Session-based result caching to improve response times
- **Progressive Loading**: Chunked processing for large datasets to maintain responsiveness

9.0.3 Analysis Workflow Management

The application implements a modular analysis pipeline:

1. **Data Preprocessing**: SMILES validation and standardization with error reporting
2. **Diversity Scoring**: Parallel execution of multiple scorers with progress tracking
3. **Results Aggregation**: Combined statistical analysis with significance testing
4. **Visualization Generation**: Interactive plot creation with customizable parameters
5. **Export Functionality**: CSV and high-resolution image export options

9.1 Technical Specifications

9.1.1 Dependencies and Requirements

Core Libraries:

- **RDKit** ($\geq$ 2023.03): Cheminformatics calculations and molecular manipulation
- **Streamlit** ($\geq$ 1.28): Web application framework with reactive components
- **Pandas/NumPy**: Data manipulation and numerical computing optimizations
- **Scikit-learn**: Machine learning algorithms for clustering and dimensionality reduction
- **Plotly**: Interactive visualization with WebGL acceleration
- **NetworkX**: Graph-based molecular analysis for connectivity patterns

System Requirements:

- **Python**: Version 3.10 or higher with modern asyncio support
- **Memory**: Minimum 4GB RAM (8GB+ recommended for datasets $>$ 10,000 molecules)
- **CPU**: Multi-core processor recommended for parallel fragment processing
- **Storage**: Approximately 500MB for installation, additional space for analysis outputs

9.1.2 Integration Capabilities

REINVENT4 Integration:

- **Real-time Scoring**: Integration with reinforcement learning workflows
- **Custom Alerts**: Automated diversity constraint enforcement during generation
- **Batch Processing**: High-throughput molecular generation analysis

Export Formats:

- **CSV Results**: Structured diversity metrics and molecular data
- **Publication Graphics**: High-resolution figures (PNG, SVG) with customizable styling
- **Interactive Plots**: Exportable HTML visualizations with embedded data
- **Statistical Reports**: Comprehensive analysis summaries with significance testing

9.1.3 Python Package Structure

The NaviDiv diversity analysis tool is implemented as a comprehensive Python package with a modular, object-oriented architecture. The package structure follows standard Python packaging conventions while maintaining clear separation of concerns across different analysis domains.

9.1.4 Core Package Organization

Listing 5: NaviDiv Package Structure

```
navidiv/
|-- __init__.py                # Package initialization
|-- utils.py                   # Utility functions and helpers
|-- scorer.py                  # Abstract base scorer class
```

```
|-- diversity/                   # Core diversity calculation modules
|   |-- __init__.py
|   |-- diversity.py             # Main diversity metrics implementation
|   +-- utils.py                 # Diversity-specific utilities
|-- fragment/                    # Fragment-based analysis
|   |-- __init__.py
|   |-- fragment_scorer.py       # BRICS fragmentation scorer
|   |-- fragment_scorer_matching.py # MCS-based fragment matching
|   |-- ring_scorer.py           # Ring system analysis
|   |-- fg_scorer.py             # Functional group scorer
|   +-- utils.py                 # Fragment utilities
|-- scaffold/                    # Scaffold-based analysis
|   |-- __init__.py
|   |-- Scaffold_scorer.py       # Main scaffold extraction
|   |-- Scaffold_GNN.py          # GNN-derived scaffolds
|   +-- utils.py                 # Scaffold utilities
|-- simlarity/                   # Similarity-based clustering
|   |-- __init__.py
|   |-- cluster_similarity_scorer.py # Clustering algorithms
|   +-- orginal_similarity_scorer.py # Basic similarity metrics
|-- stringbased/                 # String pattern analysis
|   |-- __init__.py
|   +-- Ngram_scorer.py          # N-gram pattern analysis
|-- app_utils/                   # Streamlit application utilities
|   |-- __init__.py
|   |-- action_func.py           # Main application actions
|   |-- description.py           # UI descriptions and help text
```

```
|   |-- file_name_registry.py      # File naming management
|   |-- plot_generated_molecules.py # Molecular visualization
|   +-- plot_results.py            # Results plotting
+-- reinvent/                       # REINVENT4 integration
    |-- __init__.py
    |-- run_staged_learning_2.py    # RL training integration
    +-- InputGenerator.py           # Configuration generation
```

9.1.5 Core Module Descriptions

Base Framework (`scorer.py`):

- Abstract `BaseScore` class defining the common interface
- Statistical analysis methods for fragment-score correlations
- Memory-efficient data structures for large-scale analysis
- Parallel processing capabilities for computational efficiency

Diversity Module (`diversity/`):

- Core diversity metrics implementation (HamDiv, IntDiv, etc.)
- Distance matrix calculations using molecular fingerprints
- Tanimoto similarity and clustering algorithms
- Ring system and functional group identification utilities

Fragment Analysis (`fragment/`):

- `FragmentScorer`: BRICS-based molecular fragmentation
- `FragmentMatchScorer`: Maximum Common Substructure (MCS) matching

- `RingScorer`: Ring system extraction and analysis
- `FGScorer`: Functional group identification and scoring

Scaffold Analysis (`scaffold/`):

- `Scaffold_scorer`: Classical Bemis-Murcko scaffold extraction
- `ScaffoldGNNScorer`: Machine learning-derived scaffolds
- Multiple scaffold abstraction levels (full, wireframe, elemental)
- Graph-based scaffold comparison algorithms

Similarity Analysis (`simlarity/`):

- `ClusterSimScorer`: Configurable molecular clustering
- Multiple similarity metrics (Tanimoto, Dice, Euclidean)
- Hierarchical and density-based clustering algorithms
- Fingerprint generation and comparison utilities

String Analysis (`stringbased/`):

- `NgramScorer`: SMILES n-gram extraction and analysis
- Configurable n-gram sizes for different granularity levels
- Levenshtein distance calculations for string similarity
- Pattern frequency analysis with statistical significance testing

9.1.6 Application Layer (`app_utils/`)

The web application layer provides a user-friendly interface built on Streamlit:

Core Application Functions:

- `action_func.py`: Main application workflows (t-SNE, scoring, analysis)
- `description.py`: User interface descriptions and documentation
- `file_name_registry.py`: Systematic file naming and organization
- `plot_generated_molecules.py`: Interactive molecular visualization
- `plot_results.py`: Results plotting and statistical visualization

9.1.7 Integration Layer (`reinvent/`)

REINVENT4 integration modules enable seamless incorporation with reinforcement learning workflows:

Integration Components:

- `run_staged_learning_2.py`: Modified REINVENT4 training loop with diversity constraints
- `InputGenerator.py`: Automated configuration file generation for diversity-aware runs
- Custom scoring components that integrate with REINVENT4's component system
- Real-time diversity monitoring during molecular generation

9.1.8 Configuration and Installation

The package is configured using modern Python packaging standards:

Listing 6: Package Configuration (pyproject.toml)

```
[project]
name = "navidiv"
```

```
dependencies = [
  "numpy", "scipy", "pandas", "matplotlib", "rdkit",
  "streamlit", "scikit-learn", "igraph", "plotly"
]
requires-python = ">=3.10"

[project.optional-dependencies]
reinvent = [
  "hydra-core", "mmpdb==2.1", "pydantic", "tensorboard",
  "xxhash", "python-dotenv", "molvs", "pathos", "chemprop==1.5.2"
]
dev = [
  "ruff", "mypy", "pytest", "pytest-cov", "sphinx", "furo"
]
```

Installation Options:

- **Standard Installation**: `pip install -e .` for core diversity analysis
- **REINVENT Integration**: `pip install -e .[reinvent]` for full RL capabilities
- **Development Setup**: `pip install -e .[dev]` for contributing and testing

9.1.9 API Design Philosophy

The package follows consistent design principles:

Inheritance Hierarchy: All scorer classes inherit from `BaseScore`, ensuring:

- Consistent method signatures across all diversity metrics
- Standardized input/output formats for interoperability
- Common statistical analysis capabilities

- Unified result export and visualization interfaces

Modular Architecture:

- Each analysis type (fragment, scaffold, similarity) is self-contained
- Cross-module dependencies minimized for maintainability
- Plugin-style architecture allows easy addition of new metrics
- Configuration-driven behavior for flexible parameter tuning

Performance Optimization:

- Vectorized operations using NumPy and Pandas
- Parallel processing for computationally intensive operations
- Memory-efficient data structures for large molecular datasets
- Caching mechanisms for repeated calculations

This modular, extensible architecture enables both standalone diversity analysis and seamless integration with existing molecular design workflows, making NaviDiv suitable for both research applications and production deployments.

References

(1) Kruger, F.; Stiefl, N.; Landrum, G. A. rdScaffoldNetwork: The Scaffold Network Implementation in RDKit. *Journal of Chemical Information and Modeling* **2020**, *60*, 3331–3335.

(2) Schaufelberger, L.; Blaskovits, J. T.; Laplaza, R.; Jorner, K.; Corminboeuf, C. Inverse Design of Singlet-Fission Materials with Uncertainty-Controlled Genetic Optimization. *Angewandte Chemie* **2025**, *137*, e202415056.

(3) Worakul, T.; Laplaza, R.; Terence Blaskovits, J.; Corminboeuf, C. Generative design of singlet fission materials leveraging a fragment-oriented database. *Chem. Sci.* **2025**, *16*, 17956–17969.
